# Genotypic and allelic frequencies of progressive rod‐cone degeneration and other main variants associated with progressive retinal atrophy in Italian dogs

**DOI:** 10.1002/vro2.77

**Published:** 2023-11-23

**Authors:** Sara Ghilardi, Mara Bagardi, Stefano Frattini, Giulia E. Barbariga, Paola G. Brambilla, Giulietta Minozzi, Michele Polli

**Affiliations:** ^1^ Department of Veterinary Medicine and Animal Sciences—DIVAS University of Milan Lodi Italy; ^2^ Vetogene Laboratory—ENCI Servizi SRL Milan Italy

## Abstract

**Background:**

Progressive retinal atrophy (PRA) is a group of canine inherited retinal disorders affecting up to 100 breeds. Genetic tests are available. The aim of this study was to retrospectively evaluate the genetic variants associated with PRA among dogs residing in Italy.

**Methods:**

Genetic data of 20 variants associated with different forms of PRA were collected through DNA tests over a 10‐year period for several dog breeds in the Italian canine population. Allelic and genotypic frequencies were calculated.

**Results:**

A total of 1467 DNA tests were conducted for 1180 dogs. Progressive rod‐cone degeneration (PRCD) was the most tested form of PRA, with 58.15% (*n* = 853) of the DNA tests. Among the widespread breeds in Italy, Labrador retrievers and toy poodles showed a prevalence of heterozygous carriers higher than 15%. Among the others, 175 DNA tests for golden retrievers (GR) showed a prevalence of heterozygous carriers of 13.04% (*n* = 12) for GR‐PRA1 and 8.43% (*n* = 7) for GR‐PRA2. The zwergschnauzer breed was tested for the type B and/or the type B1 forms of PRA with 25.32% (*n* = 20) heterozygous carriers and 0%, respectively.

**Conclusion:**

The study offers an overview of the prevalence of PRCD and other PRA forms within some of the most popular breeds in Italy.

## INTRODUCTION

Progressive retinal atrophy (PRA) is a heterogeneous group of inherited retinal disorders affecting up to 100 canine breeds and has been shown to be homologous with retinitis pigmentosa in humans.^1^, ^2^, ^3^, ^4^, ^5^ The disease is characterised by a primary degeneration of rod photoreceptors followed by the degeneration of cones. This initially causes night blindness, which then progresses to severe visual dysfunction under both dim and bright light; finally, the degeneration of cones can lead to total blindness.^3^ Some genetic variants responsible for PRA can lead to rod degeneration only, while others can involve cones as well; however, an earlier and more severe loss of rods represents the peculiar PRA phenotype.^1^ Affected dogs show changes in the fundus in an ophthalmologic examination, such as hyperreflectivity of the tapetum, attenuation of retinal blood vessels and atrophy of the optic disk. At the early stage of the disease, electroretinography can be useful to distinguish PRA from other ocular diseases.^1^, ^6^ Development of secondary cataracts in the late stage of the disease is often subsequently diagnosed.^1^, ^7^

While clinical signs are similar among various forms of PRA, the aetiology, age of onset and rate of progression vary between and within breeds.^8^ The early‐onset form manifests during the postnatal retinal differentiation process and results from retinal degeneration or abnormal/interrupted retinal development; the progression rate of this form is fast and affected dogs usually manifest clinical signs at a young age. Late‐onset PRA, on the contrary, is characterised by a retinal degeneration that starts after the maturation of the retina itself is complete; it shows a slow progression rate and clinical signs may not be manifest until later in life. Usually, it occurs because of defects in the correct maintenance of photoreceptors’ normal function.^1^

Various modes of inheritance have been described for PRA including autosomal recessive, autosomal dominant and X‐linked; the most common mode of inheritance is recessive.^2^, ^3^ Over 20 genes have been identified in PRA across different breeds with a wide range of genetic alleles reported.^9^, ^10^, ^11^, ^12^, ^13^, ^14^, ^15^, ^16^, ^17^ Genetic tests are available for the detection of various forms of PRA and are able to detect affected/at‐risk dogs, heterozygous carriers and clear subjects.^18^

The principal aim of this study was to retrospectively evaluate allele and genotype frequencies of different PRA‐associated variants in Italian dogs over a 10‐year period. This included assessing, for each dog, the genotypic heterozygosis or homozygosis, in order to provide a prevalence for the clear dogs, as well as of the affected/at‐risk subjects and the heterozygous carriers.

## MATERIALS AND METHODS

### Ethical approval statement

All animals included in the study were owned by private owners or breeders, who provided written consent. Blood samples were collected by an authorised veterinarian.

### Description of the study

This was a retrospective observational study. All DNA tests and associated results for the detection of PRA were provided by a commercial laboratory, Vetogene Laboratory (VL), between 2010 and 2021. The DNA tests were conducted on samples from dogs from all over Italy. The VL is one of the official reference laboratories for the execution of DNA tests for the Italian Kennel Club Ente Nazionale Cinofilia Italiana (ENCI). The VL received blood samples from dog owners, veterinarians and breeders who wanted to conduct the DNA test for PRA. Blood samples were officially collected by veterinarians into EDTA tubes and stored at +4°C for a maximum of 4–5 days prior to examination; each sample was accompanied by a certification from the veterinarian. Some of the dogs were tested for only one form of PRA, while others were tested for two or three forms of the condition. For each dog, breed and DNA test results (clear, heterozygous carrier, affected/at risk) were recorded.

### DNA extraction and analysis

The DNA extraction was obtained from 100 to 200 μL of the blood samples using the commercial Qiagen DNeasy Blood & Tissue kit (Qiagen, Hilden, Germany). Table 1 lists the 20 forms of PRA, with an associated publication reference that describes the procedures carried out by the VL to identify the PRA mutations for each specific gene. All samples were analysed by real‐time PCR methods for each specific gene mutation, as listed in Table 1. Each genotype was generated with a single test, performing a single amplification for each mutation. The genotyping was validated directly on the sequence, looking at the mutation using the Sanger sequencing method.

**TABLE 1:** Identified genes for progressive retinal atrophy (PRA) in dogs, description of the disease and list of affected/at‐risk breeds.

OMIA variant ID	OMIA phenotype ID	Gene name ( canine chromosome)	Tested variant^a^	Disease and transmission	Affected/at‐risk breed(s)	Publication reference
76	OMIA001298‐9615	*PRCD* (CFA9)	g.4188663C>T	PRCD (Progressive rod‐cone degeneration)^b^	Various number of breed and mixed breeds	^14^
575	OMIA001572‐9615	*SLC4A3* (CFA37)	g.26145752_26145753insC	Golden retriever PRA1^b^	Golden retriever	^19^
949	OMIA001984‐9615	*TTC8* (CFA8)	g.60090186del	Golden retriever PRA2^b^	Golden retriever	^20^
282	OMIA000882‐9615	*PDE6B* (CFA3)	g.91747713C>T	RCD1 (Rod‐cone dysplasia)^b^	Irish setter	^21^
528	OMIA001674‐9615	*PDE6B* (CFA3)	g.91747713C>T	RCD1b^b^	American Staffordshire terrier	^22^
*710*	OMIA:001260‐9615	*RD3* (CFA7)	Insertion_22 bp	RCD2^b^	Collie	^11^
475	OMIA001314‐9615	*PDE6A* (CFA4)	g.59145362del	RCD3^b^	Cardigan Welsh corgi	^12^
583	OMIA:001575‐9615	*C2orf71* (CFA37)	c.3149_3150insC	RCD4^b^	Gordon setter, Irish setter	^23^
359	OMIA001876‐9615	*SAG* (CFA25)	g.44843440T>C	PRA^b^	Basenji	^24^
547	OMIA001977‐9615	*CNGA1* (CFA13)	g.43831897_43831900del	PRA^b^	Shetland sheepdog	^25^
918	OMIA000830‐9615	*CNGB1* (CFA2)	g.58622673_58622675delinsCTAGCTAC	PRA^b^	Papillon, phalène dog	^26^
699	OMIA001432‐9615	*RPGRIP1* (CFA15)	g.18332036_18332037ins	CORD1 (Cone‐rod dystrophy)^b^	Miniature longhaired dachshund	^27^
634	OMIA001455‐9615	*NPHP4* (CFA5)	g.59912991_59913168del	CORD2^b^	Standard wirehaired dachshund	^28^
574	OMIA001521‐9615	*CCDC66* (CFA20)	g.33745452_33745453insT	Generalised PRA^b^	Schapendoes, Portuguese water dog	^29^, ^30^
29	OMIA001346‐9615	*RHO* (CFA20)	g.5637394G>C	Autosomal dominant PRA^c^	English mastiff, bullmastiff	^13^
480	OMIA000831‐9615	*RPGR* (CFAX)	g.33126490_33126494del	X‐linked PRA	Siberian husky, samoyed	^17^
641	OMIA001523‐9615	*COL9A2* (CFA15)	Deletion_1267 bp	Oculoskeletal dysplasia	Labrador retriever, samoyed	^31^
		*PDC* (CFA7)	c.244C>G p.Arg82Gly	PRA type A	Miniature schnauzer	^32^
1068	OMIA:001311‐9615	*PPT1* (CFA15)	g.2874661_2875048con2877563_2877607inv	PRA type B	Miniature schnauzer	^33^
1170	OMIA:001311‐9615	*HIVEP3* (CFA15)	g.1432293G>A	PRA type B1	Miniature schnauzer	^33^

*Note*: Only PRA forms evaluated in the present study are reported. Online Mendelian Inheritance in Animals (OMIA) identification numbers are provided for each phenotype and variant where available.

^a^ All variant positions are referred to the CanFam3.1 genome build, except for X‐linked PRA, which refers to the ROS_Cfam 1.0 genome build.

^b^ Autosomal recessive.

^c^ Autosomal dominant.

### Statistical analysis

Analyses were conducted using SAS 9.4 software (SAS Inc.). Initially, frequencies of clear, heterozygous carriers and affected/at‐risk dogs were recorded according to the breed. Afterwards, only the PRA forms for groups of breeds that counted more than 15 subjects were analysed. For these, allelic and genotypic frequencies were evaluated through the PROC FREQ and PROC ALLELE analyses offered with SAS.

## RESULTS

A total of 1467 DNA tests for PRA were conducted for 1180 dogs: 81.78% (*n* = 965) were tested for one form of PRA, 12.12% (*n* = 143) for two forms and 6.10% (*n* = 72) for three forms. Absolute and relative frequencies of clear, heterozygous carriers and affected/at‐risk dogs divided according to the PRA form are given in Table 2. Only frequencies for the seven forms with more than 45 DNA tests are reported for statistical relevance (further results are given in Table S1). In 85.07% of the tested dogs, the test result was clear for the gene (*n* = 1248), with 13.84% heterozygous carriers (*n* = 203) and 1.09% of dogs deemed affected/at risk (*n* = 16).

**TABLE 2:** Absolute and relative frequencies of clear, heterozygous carriers and affected/at‐risk Italian dogs of the forms of progressive retinal atrophy (PRA) for which more than 45 DNA test results were collected.

Gene	Clear	Heterozygous/carrier	Affected/at risk	Total
Progressive Rod‐Cone Degeneration	717	128	8	853
	84.06%	15.01%	0.94%	
Golden retriever PRA1	80	12	0	92
	86.96%	13.04%	0%	
Golden retriever PRA2	76	7	0	83
	91.57%	8.43%	0%	
Rod‐cone dysplasia ‐ RCD1	27	16	3	46
	58.7%	34.78%	6.52%	
Cone‐rod dystrophy ‐ CORD1	37	10	2	49
	75.51%	20.41%	4.08%	
PRA type B	58	20	1	79
	73.42%	25.32%	1.27%	
RCD4	90	5	1	96
	93.75%	5.21%	1.04%	
Other^a^	163	5	1	169
	96.45%	2.95%	0.60%	
Total	1248	203	16	1467
	85.07%	13.84%	1.09%	

^a^ Forms of PRA with less than 45 DNA tests included PRA type A (*n* = 31), CORD2 (*n* = 30), RCD3 (*n* = 23), X‐linked PRA (*n* = 19), PRA (*CNGA1*) (*n* = 14), RCD2 (*n* = 14), PRA type B1 (*n* = 12), oculoskeletal dysplasia (*n* = 9), PRA (*CNGB1*) (*n* = 6), RCD1b (*n* = 5), PRA (*SAG*) (*n* = 2), generalised PRA (*n* = 2) and autosomal dominant PRA (*n* = 2) (see Table S1).

Progressive rod‐cone degeneration (PRCD) was the most common test performed for PRA, with 58.15% (*n* = 853) of the DNA tests. The rod‐cone dysplasia (RCD)1 form presented with the highest number of heterozygous carriers (34.78%) and affected/at‐risk subjects (6.52%) among the PRA forms. The RCD1 was only tested in the Irish setter, given a previous study.^21^

Fifty‐five breeds were tested and, in line with breed‐distribution data reported for Italy by ENCI, the five most represented breeds in our population were golden retriever (19%), toy poodle (15%), zwergschnauzer (9%), Labrador retriever (9%) and the Australian shepherd dog (8%). The remaining dogs belonged to 50 different breeds, with each of them having a prevalence lower than 5%. A detailed analysis of the five breeds showed that PRCD was the PRA form with the highest number of heterozygous carriers (20% toy poodle, 15.79% Labrador retriever and 3.33% Australian shepherd dog). The samples from golden retriever dogs showed an absence of the *PRCD* risk allele, with all 104 tested subjects being clear. The zwergschnauzer‐related samples were not tested for PRCD; all subjects in this breed were tested for the type B and/or the type B1 forms of PRA, with 25.32% heterozygous carriers for type B and no positive results for type B1.

Progressive rod‐cone degeneration was tested in 34 different breeds. Table 3 summarises the number of clear, heterozygous carriers and PRCD‐affected/at‐risk dogs for each breed, considering only the breeds in which more than 15 dogs were tested (*n* = 9). Six of these breeds presented with heterozygous carrier percentages higher than 15%. The Australian cattle dog had a high number of PRCD heterozygous carriers (33.90%) and affected/at‐risk subjects (3.39%) compared with other breeds. A further 20 breeds were tested for PRCD (*n* = 160) with none positive for heterozygous carrier status or affected/at‐risk dogs; they are not reported in Table 3.

**TABLE 3:** Absolute and relative frequencies of clear, heterozygous carriers and progressive rod‐cone degeneration (PRCD)‐affected/at‐risk Italian dogs, divided according to breed.

	Clear	Heterozygous carrier	Affected/at risk	Total
**Toy poodle** ^a^	137	35	3	175
	78.29%	20.00%	1.71%	
**Labrador retriever** ^a^	111	21	1	133
	83.46%	15.79%	0.75%	
Australian shepherd dog^a^	116	4	0	120
	96.67%	3.33%	0%	
**English cocker spaniel**	56	20	1	77
	72.73%	25.97%	1.30%	
**Australian cattle dog**	37	20	2	59
	62.71%	33.90%	3.39%	
Standard poodle	46	6	0	52
	88.46%	11.54%	0%	
Bolognese dog	15	1	0	16
	93.75%	6.25%	0%	
**Miniature poodle**	12	4	0	16
	75.00%	25.00%	0%	
**Nova Scotia duck tolling retriever**	9	7	0	16
	56.25%	43.75%	0%	
Other breeds^b^	18	10	1	29
	62.07%	34.48%	3.45%	
Total	557	128	8	693
	80.36%	18.48%	1.16%	

*Note*: Only breeds that counted more than 15 subjects are reported. In bold, the breeds that presented with a percentage of heterozygous carriers higher than 15%.

^a^ Dog breeds that are among the five most popular breeds reported in Italy.

^b^ Breeds that counted fewer than 15 subjects include Portuguese water dog (*n* = 11), Entlebucher mountain dog (*n* = 8), Karelian bear dog (*n* = 6), Kai (*n* = 3) and Lapponian herder (*n* = 1).

The DNA tests for golden retriever‐(GR)‐PRA1 and GR‐PRA2 were only conducted on samples from golden retrievers because the disease is only reported in this breed.^19^, ^20^ Ninety‐two dogs were tested for GR‐PRA1 and 13.04% (*n* = 12) were heterozygous carriers of the *SLC4A3* gene mutation. For GR‐PRA2, 83 dogs were tested and 8.43% (*n* = 7) were heterozygous carriers of the *TTC8* risk allele. None of the tested golden retrievers were considered to be affected by/at risk of developing either disease.

The allelic frequencies for the most widespread forms of PRA are reported in Table S2.

## DISCUSSION

To the best of the authors’ knowledge, this is the first study of the allelic and genotypic frequencies of PRA for dogs drawn from the Italian canine population over a 10‐year period. An interesting result is the large number of DNA tests conducted, indicating that genetic testing is considered a useful tool by veterinarians and breeders to detect heterozygous carriers and affected/at‐risk subjects. The results of our study detected a high percentage of PRA risk allele carriers in the Italian population (13.84% of heterozygous carriers), a percentage that increased when analysing only the Irish setter for the RCD1 form (34.78% of heterozygous carriers). These frequencies represent a great concern, especially because most of the DNA tests were conducted on dogs, which are used as breeding animals. The RCD1 is a late‐onset form of PRA; however, it should be suggested to breeders to conduct a DNA test on Irish setters in the first years of life because dogs are used for reproduction from a young age (2 years old), so the mating of untested pairs represents a great risk.

The results showed that only 1.09% of the tested dogs were affected by/at risk of manifesting PRA. This result may be because veterinarians and breeders do not require a DNA test in a dog with a clinical diagnosis of PRA. The Italian Kennel Club demands a clinical certification of PRA exemption in some breeds to allow the dog into specific competitions; consequently, these dogs are presented for an ophthalmological evaluation from a young age (6–8 months) and could possibly be diagnosed with early‐onset forms of PRA. However, an early ophthalmological examination does not exclude the late‐onset forms of PRA, such as PRCD, which appears at an adult‐old age. Therefore, DNA testing in these cases is imperative to identify heterozygous carriers and affected/at‐risk dogs. Thus, the limited use of DNA tests in late‐onset forms of PRA could be the reason why PRCD is the most widespread PRA form in our population, along with GR‐PRA1, GR‐PRA2 and CORD1. Progressive rod‐cone degeneration is the most widespread form of PRA mainly because it affects several different breeds,^14^, ^15^ while another explanation of the diffusion of GR‐PRA1 and GR‐PRA2 lies in the large numbers of golden retrievers present and tested in Italy.

In recent years, there have been few published studies on the genotypic and allelic frequencies of canine PRA in other countries.^34^, ^35^, ^36^, ^37^ One genetic study,^38^ with sample sizes consisting of millions of dogs from 150 countries, reported results on 250 genetic disease‐associated variants in the general dog population. It included PRA‐related statistics that varied between purebred and mixed breed dogs. The allele frequency for the PRCD allele in 16,825 Labrador retrievers was 7.2%, which is in line with the reported 15.79% carrier frequency in our samples from Labrador retrievers. The allelic frequency for PRCD in Australian shepherd dogs was 0.38% (tested for 2293 dogs), while our study identified a lower incidence of that risk allele (0.02%) although the population size in our study was only 120 dogs (Table S2). The allelic frequency in 94 toy poodles was estimated to be 2.1%,^38^ compared with 11.7% in our sample of 175 dogs from the Italian population. In other breeds, when the number of animals tested was higher, as in the case of the standard poodle, the frequency of the risk allele was more comparable. The PRCD was 0.5% in 4197 standard poodles,^38^ compared with 5.8% in 52 dogs from the Italian population. The frequency of the PRCD allele in the English cocker spaniel was estimated to be 9.5% based on 579 dogs,^38^ compared with our result of 14.3% for 77 dogs in the Italian cohort.

In conclusion, our study offers an overview of the genotypic and allelic frequencies of various forms of PRA for a cohort of dogs in Italy. The results suggest that the PRA genetic test may be considered a useful tool by veterinarians and breeders to identify affected/at risk and heterozygous carriers among the canine population. Performing the DNA test in young subjects before the dogs are used in breeding programmes is desirable in order to improve dogs’ health and welfare through the implementation of specific breeding schemes.

## AUTHOR CONTRIBUTIONS

*Conceptualisation*: Michele Polli, Sara Ghilardi and Paola G. Brambilla. *Data curation*: Sara Ghilardi. *Formal analysis*: Giulia E. Barbariga. *Funding acquisition*: Michele Polli. *Sampling*: Michele Polli and Stefano Frattini. *Methodology*: Michele Polli and Giulietta Minozzi. *Writing original draft*: Michele Polli, Sara Ghilardi and Mara Bagardi. *Writing, review and editing*: Paola G. Brambilla, Sara Ghilardi, Giulietta Minozzi and Michele Polli. All authors read and agreed to the published version of the manuscript.

## CONFLICTS OF INTEREST STATEMENT

Stefano Frattini is an employee of the Vetogene Laboratory. The laboratory had no role in the design of the study, analysis and interpretation of the data, or the preparation of the article. The other authors declare they have no conflicts of interest.

## ETHICS STATEMENT

The authors confirm that the ethical policies of the journal, as noted on the journal's author guidelines page, have been adhered to. No ethical approval was required because the study analysed test data provided by the laboratory, using samples derived from general clinical practice, subject to permission to release the test results from the dog owners.

## Supporting information

Supporting Information

## Data Availability

Data analysed and described in this study are available from the corresponding authors upon reasonable request.
