# Supplementary material for: Genotypic and allelic frequencies of progressive rod‐cone degeneration and other main variants associated with progressive retinal atrophy in Italian dogs
Source: Vet Rec Open. 2023 Nov 23;10(2):e77. doi: 10.1002/vro2.77 (PMC10665785; doi:10.1002/vro2.77)
Supplement: Supplementary file 1 — Supporting Information [file VRO2-10-e77-s001.docx]

**Supporting Information**

## **Table S1 Absolute and relative frequencies of clear, heterozygous carriers and affected/at risk Italian dogs for each analyzed form of Progressive retinal atrophy (PRA)**

|  | Clear | Heterozygous carrier | Affected/at risk | Total |  |  | Healthy | Carrier | Affected | Total |
| --- | --- | --- | --- | --- | --- | --- | --- | --- | --- | --- |
| PRA Type A  (Progressive retinal atrophy) | 31 | 0 | 0 | 31 |  | OSD  (Oculoskeletal dysplasia) | 9 | 0 | 0 | 9 |
|  | 100% | 0% | 0% |  |  |  | 100% | 0% | 0% |  |
| CORD2  (Cone-rod dystrophy) | **25** | **4** | **1** | **30** |  | PRA *(CNGB1)* | 6 | 0 | 0 | 6 |
|  | **83.33%** | **13.33%** | **3.33%** |  |  |  | 100% | 0% | 0% |  |
| RCD3  (Rod-cone dyspasia) | 23 | 0 | 0 | 23 |  | RCD1b | 5 | 0 | 0 | 5 |
|  | 100% | 0% | 0% |  |  |  | 100% | 0% | 0% |  |
| XLPRA  (X-linked progressive retinal atrophy) | 19 | 0 | 0 | 19 |  | Generalised PRA | 2 | 0 | 0 | 2 |
|  | 100% | 0% | 0% |  |  |  | 100% | 0% | 0% |  |
| RCD2 | 14 | 0 | 0 | 14 |  | ADPRA  (Autosomal dominant progressive retinal atrophy) | 2 | 0 | 0 | 2 |
|  | 100% | 0% | 0% |  |  |  | 100% | 0% | 0% |  |
| PRA *(CNGA1)* | 14 | 0 | 0 | 14 |  | **PRA *(SAG)*** | **1** | **1** | **0** | **2** |
|  | 100% | 0% | 0% |  |  |  | **50%** | **50%** | **0%** |  |
| PRA Type B1 | 12 | 0 | 0 | 12 |  | Other* | 1085 | 198 | 15 | 1298 |
|  | 100% | 0% | 0% |  |  |  | 84.50% | 15.42% | 0.00% |  |
|  |  |  |  |  |  | **Total** | **1248** | **203** | **16** | **1467** |
|  |  |  |  |  |  |  | **85.07%** | **13.84%** | **1.09%** |  |

In bold, forms of PRA which present the gene mutation in the population (heterozygous carriers and/or affected/at risk dogs).

* Forms of PRA with more than 45 DNA tests (See Table 2 in main article).

## **Table S2 Allelic frequencies for the mutations in the genes responsible for Progressive rod-cone degeneration, Progressive retinal atrophy Type B, Cone-rod dystrophy CORD1 and CORD2, divided according to the breed**

| Gene | Breed/Group | Mutated allele | | Normal allele | |
| --- | --- | --- | --- | --- | --- |
|  |  | **Frequency** | **Confidence interval** | **Frequency** | **Confidence interval** |
| PRCD  (Progressive rod-cone degeneration) | Poodle  n = 243 | 0.105 | 0.078-0.133 | 0.896 | 0.867-0.922 |
|  | Toy poodle*  n = 175 | 0.117 | 0.083-0.151 | 0.883 | 0.849-0.917 |
|  | Standard poodle*  n = 52 | 0.058 | 0.031-0.250 | 0.942 | 0.894-0.981 |
|  | Miniature poodle*  n = 16 | 0.125 | 0.031-0.250 | 0.875 | 0.750-0.969 |
|  | Labrador retriever  n = 133 | 0.087 | 0.053-0.124 | 0.914 | 0.876-0.947 |
|  | Australian shepherd  n = 120 | 0.017 | 0.004-0.033 | 0.983 | 0.967-0.996 |
|  | English cocker spaniel  n = 77 | 0.143 | 0.091-0.195 | 0.857 | 0.805-0.909 |
|  | Australian cattle dog  n = 59 | 0.203 | 0.136-0.280 | 0.797 | 0.720-0.864 |
| PRA Type B  (Progressive retinal atrophy) | Zwergschnauzer  n = 79 | 0.139 | 0.089-0.196 | 0.861 | 0.804-0.911 |
| CORD1  (Cone-rod dystrophy) | Dachshund  n = 35 | 0.171 | 0.092-0.263 | 0.829 | 0.737-0.908 |
| CORD2 | Standard Dachshund  n = 27 | 0.074 | 0.000-0.167 | 0.926 | 0.833-1.000 |

* subsets of the total tested population.
