# Supplementary material for: Genotypic and allelic frequencies of progressive rod‐cone degeneration and other main variants associated with progressive retinal atrophy in Italian dogs
Source: Vet Rec Open. 2023 Nov 23;10(2):e77. doi: 10.1002/vro2.77 (PMC10665785; doi:10.1002/vro2.77)
Supplement: Supplementary file 2 [file VRO2-10-e77-s002.pdf]

Supporting Information

Table S1 Absolute and relative frequencies of clear, heterozygous carriers and affected/at risk Italian dogs for each analyzed form of Progressive retinal atrophy (PRA)

	Clear	Heterozygous carrier	Affected/at risk	Total			Healthy	Carrier	Affected	Total
PRA Type A (Progressive retinal atrophy)	31	0	0	31		OSD (Oculoskeletal dysplasia)	9	0	0	9
	100%	0%	0%				100%	0%	0%	
**CORD2 (Cone-rod dystrophy)**	**25**	**4**	**1**	**30**		PRA *(CNGB1)*	6	0	0	6
	**83.33%**	**13.33%**	**3.33%**				100%	0%	0%	
RCD3 (Rod-cone dyspasia)	23	0	0	23		RCD1b	5	0	0	5
	100%	0%	0%				100%	0%	0%	
XLPRA (X-linked progressive retinal atrophy)	19	0	0	19		Generalised PRA	2	0	0	2
	100%	0%	0%				100%	0%	0%	
RCD2	14	0	0	14		ADPRA (Autosomal dominant progressive retinal atrophy)	2	0	0	2
	100%	0%	0%				100%	0%	0%	
PRA *(CNGA1)*	14	0	0	14		**PRA *(SAG)***	**1**	**1**	**0**	**2**
	100%	0%	0%				**50%**	**50%**	**0%**	
PRA Type B1	12	0	0	12		Other*	1085	198	15	1298
	100%	0%	0%				84.50%	15.42%	0.00%	
						**Total**	**1248**	**203**	**16**	**1467**
							**85.07%**	**13.84%**	**1.09%**	

In bold, forms of PRA which present the gene mutation in the population (heterozygous carriers and/or affected/at risk dogs).
* Forms of PRA with more than 45 DNA tests (See Table 2 in main article).

Table S2 Allelic frequencies for the mutations in the genes responsible for Progressive rod-cone degeneration, Progressive retinal atrophy Type B, Cone-rod dystrophy CORD1 and CORD2, divided according to the breed

Gene	Breed/Group	Mutated allele		Normal allele	
		Frequency	Confidence interval	Frequency	Confidence interval
PRCD (Progressive rod-cone degeneration)	Poodle n = 243	0.105	0.078-0.133	0.896	0.867-0.922
	Toy poodle* n = 175	0.117	0.083-0.151	0.883	0.849-0.917
	Standard poodle* n = 52	0.058	0.031-0.250	0.942	0.894-0.981
	Miniature poodle* n = 16	0.125	0.031-0.250	0.875	0.750-0.969
	Labrador retriever n = 133	0.087	0.053-0.124	0.914	0.876-0.947
	Australian shepherd n = 120	0.017	0.004-0.033	0.983	0.967-0.996
	English cocker spaniel n = 77	0.143	0.091-0.195	0.857	0.805-0.909
	Australian cattle dog n = 59	0.203	0.136-0.280	0.797	0.720-0.864
PRA Type B (Progressive retinal atrophy)	Zwergschnauzer n = 79	0.139	0.089-0.196	0.861	0.804-0.911
CORD1 (Cone-rod dystrophy)	Dachshund n = 35	0.171	0.092-0.263	0.829	0.737-0.908
CORD2	Standard Dachshund n = 27	0.074	0.000-0.167	0.926	0.833-1.000

* subsets of the total tested population.
